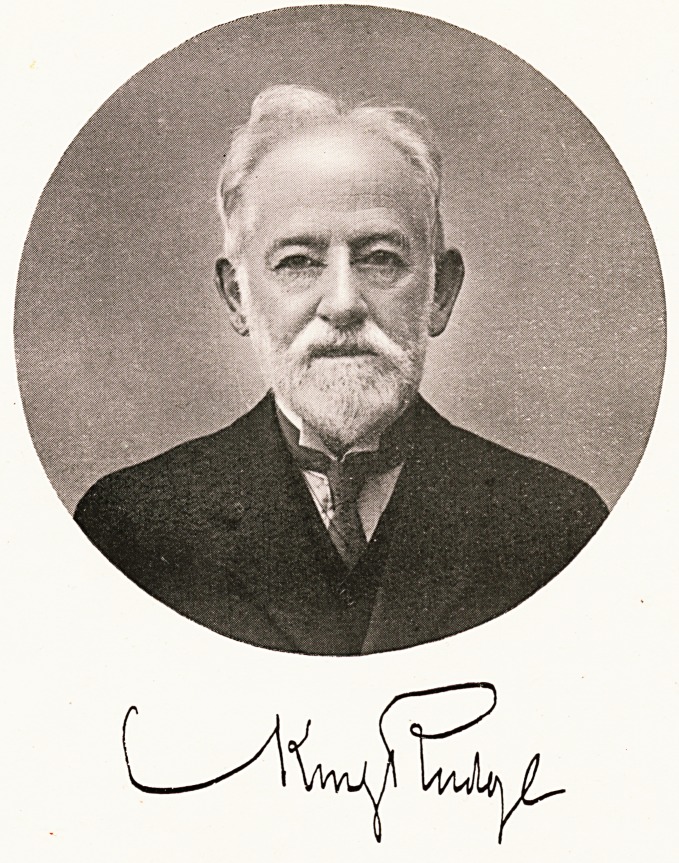# Charles King Rudge

**Published:** 1926

**Authors:** 


### Obituary.

### CHARLES KING RUDGE, M.R.C.S., L.R.C.P.

CHARLES KING RUDGE, whose death occurred on October 24th, 1926, was born at Haverfordwest, Pembrokeshire, in 1846. He came of an ancient Staffordshire family, a branch of which settled in Gloucestershire in the seventeenth century. In 1867 he entered as a medical student at the Bristol Royal Infirmary, where he gained the Suple Gold Medal for Medicine. As a student he attracted the attention of Dr. E. Long Fox and Dr. Brittan, who spoke in the highest terms of him. After obtaining the qualifications of the Conjoint Board in 1869, he was appointed Surgeon to the Bristol Dispensary and held that post for the full term of twelve years. In 1875 he started private practice in Whiteladies Road, Clifton, and continued to practise in the same house for fifty years. In 1871 Mr. Rudge married Louisa Maria, younger daughter of Richard Hughes, F.R.C.P., who predeceased him. He left issue, five sons and three daughters, to whom we offer our sincere ympathy.

Of a singularly quiet and unassuming disposition, Mr. Rudge was none the less full of varied interests and ardent in public work of many sorts. To the Bristol Medico-Chirurgical Society he gave twenty-four years of unsparing labour in a capacity which would perhaps have attracted few others among its members. When Mr. L. M. Griffiths resigned the post of Honorary Librarian to the Bristol Medical Library in 1902 Mr. Rudge consented to succeed him, and accepted re-election every year up to October, 1926. The position was not one to bring its holder into prominence, although the value to the Society and to University College, Bristol, was incalculable. Mr. Rudge was not only fond of books for their own sake ; he took an intense pride in the library which he ruled so well. He knew, of course, its history intimately ; it would be almost true, perhaps, to say that he knew the provenance of every single volume. He could point out which were held on loan from the Infirmary, or the Hospital, which had been purchased by the College, which given by the Society, and, in his eyes best of all, which had belonged to the old Bristol Medical Library. Mr. Rudge as Honorary Librarian had the anxious task of seeing his precious books moved from the present Senate Chamber to the old Blind Asylum, thence to the Racquets Courts of the Drill Hall, and finally into the worthy library designed by Sir George Oatley in the new buildings of the University, which we owe to the generosity of the Wills family.

How carefully Mr. Rudge protected the books from damage in each of these removals the state of the library shelves can testify. It was no small satisfaction to him when at last the whole collection with its multiple ownership was entrusted to the sole charge of the University. But when the gift to the University was complete he experienced for a short time one fearful misgiving. Had he helped to bury the last trace of the Bristol Medical Library ? The University, not wholly realising perhaps all that the gesture implied to Mr. Rudge, resolved that the library should be known as the Bristol Medical Library in the University of Bristol, and invited him to continue as the Honorary Medical Librarian to the University.

In 1922 the Bristol Medico-Chirurgical Society had elected Mr. Rudge to be an honorary member, the only distinction by which his services could in any measure be acknowledged, but in his inmost heart it seemed that the retention of the name of the Bristol Medical Library filled him with greater satisfaction than any personal honours could have done.

Beyond the Medical Library Mr. Rudge still found time and energy to spare outside his busy hours of practice. He had joined the Bristol Naturalists' Society in 1870, and held at various times the offices of Secretary, Librarian, Vice-President and President. He seems to have delivered no less than three presidential addresses to the Society on the occasions when he was re-elected to the Chair of President, and he contributed to the discussions three papers: "British Shore Fishes and their habits" (1888), "The Mammals of the Bristol District" (1908), and "Food of Animals and stratagems employed in obtaining it" (1913). He was a member of the Bristol Microscopical Society and of the Bristol and Gloucestershire Archæological Society.

It is impossible, however, to write of Mr. Rudge's life and activities without referring to him as a convinced and liberal-minded Churchman. He was a life-long supporter of the Church Missionary Society, and for many years acted as local Honorary Secretary to the Medical Branch of that Society, keeping in touch with the many Medical Missionaries belonging to the C.M.S. He was also a member of the Cathedral Branch of the C.E.M.S.

It often was remarked with surprise that Mr. Rudge had never been elected President of the Medico-Chirurgical Society. He had, in fact, declined nomination several times ; he used to declare at Committee meetings, when asked if he would allow himself to be nominated for President-elect, that it was quite enough work for one man to be Librarian, and he would not give up that office in order to become President. There was something more than a mere love of books (though he possessed that in a marked degree) that kept Mr. Rudge to his librarianship so firmly and so long; he was keenly alive to the importance of a good library being always at the service of every student and practitioner of medicine. His efforts were crowned by the satisfaction of knowing that the Bristol Medical Library ranked among the leading medical libraries of the kingdom.

His loss will be felt deeply wherever he was known, and particularly by the members of this Society, who owed so much to his labour.

## Figures and Tables

**Figure f1:**